# 

**Published:** 1957-01

**Authors:** 


### Contents

| | | Page |
|---|---|---|
| A DOCTOR'S VISIT TO U.S.S.R. . . | *Dr. A. M. MacLachlan* | 1 |
| THE USE AND MISUSE OF SEDATIVES AND HYPNOTIC DRUGS | *Dr. R. E. Hemphill* | 12 |
| THE PUBLIC ANALYST AND THE NEW FOOD AND DRUGS ACT (1955) . . . . . . | *Mr. E. G. Whittle* | 23 |
| DIABETES MELLITUS WITH RENAL PAPILLARY NECROSIS | *Clinical Pathological Conference* | 26 |
| CORRESPONDENCE . . . . . . . | | 29 |
| NOTES AND NEWS . . . . . . . | | 29 |
| APPOINTMENTS . . . . . . . . | | 30 |

